# Pathogenic *Nocardia*: A diverse genus of emerging pathogens or just poorly recognized?

**DOI:** 10.1371/journal.ppat.1008280

**Published:** 2020-03-05

**Authors:** Heer H. Mehta, Yousif Shamoo

**Affiliations:** Department of BioSciences, Rice University, Houston, Texas, United States of America; Nanyang Technological University, SINGAPORE

## Introduction to pathogenic *Nocardia*, a clinically relevant non-*ESKAPE* pathogen

The clear and present danger posed by the *ESKAPE* pathogens (*Enterococcus faecium*, *Staphylococcus aureus*, *Klebsiella pneumoniae*, *Acinetobacter baumanii*, *Pseudomonas aeruginosa*, and *Enterobacter* species) and their ability to evade antimicrobials is generally well appreciated [1]. There are, however, a multitude of pathogens, some more rare than others, that can be clinically challenging to diagnose and treat. *Nocardia*, a genus of aerobic actinomycetes found ubiquitously in soil and water, harbors one such group of pathogens with unique attributes and often complicated properties.

Opportunistic infections caused by *Nocardia* often afflict immunocompromised individuals like cancer patients receiving chemotherapy, individuals with AIDS, and organ transplant recipients. Although commonly considered opportunistic, among 1,000 cases of *Nocardia* infection published between 1950 and 1991, about one-third occurred in patients with no identifiable underlying predisposing conditions [2], implying that immune status is not the only factor affecting infectivity. Skin and lung are the primary infection sites for these rod shaped, gram-positive bacteria. *Nocardia* also have the ability to survive as facultative intracellular parasites within macrophages [3,4] and escape killing by human neutrophils and monocytes [5]. Infections can sometimes be largely asymptomatic, which, when coupled with the slow growth rate of *Nocardia*, makes them difficult to identify in clinical specimens [6]. Dissemination, particularly to the central nervous system (CNS), is relatively common and can be life threatening, with mortality rates as high as 85% in immunocompromised individuals [6–8]. To date, 119 species of *Nocardia* have been documented (http://www.bacterio.net/nocardia.html), with more than 40 of them being considered clinically relevant (https://www.cdc.gov/nocardiosis/health-care-workers/index.html).

## *Nocardia* infections: Difficult to identify and difficult to treat

The ability of this organism to cause infection was first recognized in 1888 [9]. However, to date, *Nocardia* has received relatively little attention as a human pathogen. The Centers for Disease Control and Prevention (CDC) estimates approximately 500 to 1000 new cases of nocardiosis infections occur every year in the United States (https://www.cdc.gov/nocardiosis/infection/index.html). This statistic is based on a study conducted in 1976 [9] but is likely to be a significant underestimate given the paucity of molecular diagnostics in that era. *Nocardia* isolates can take up to two weeks to grow on routine culture media used in clinical labs, and mixed bacterial infections can further obscure identification [3,10]. Concomitant infections in immunocompromised individuals are common and can include *Nocardia* co-infecting with other bacterial, fungal, and viral pathogens [11,12]. Tuberculosis (TB) caused by another actinomycete, *Mycobacterium tuberculosis*, is associated with chronic lung disease, and owing to the similarity in diagnosis and clinical manifestation of nocardiosis and TB, accurate identification of these two acid-fast bacilli can be difficult [13–15]. In some cases, concomitant nocardial and TB infections have also been observed and are more likely to occur in HIV-infected individuals [16]. Treatment of choice for tuberculosis is ineffective for nocardiosis, underscoring the importance of accurate diagnosis for effective treatment [13,14,17].

Diagnostic difficulty is compounded by the ability of *Nocardia* to disseminate to a variety of sites after primary infection. This makes the site and type of infection difficult to identify, often requiring invasive biopsies [18]. Time taken from identifying symptoms to making a diagnosis can vary from 3 days to as much as 30 days [19–21]. The severity of an infection sometimes leads to administration of antimicrobial treatment prior to accurate diagnosis [22], causing some infections to go undiagnosed.

Virulence in *Nocardia* has been attributed to its ability to survive and grow in a variety of human cells and evade the host immune response by production of catalase and superoxide dismutase (SOD), inhibition of phagosome-lysosome fusion, reduction of intracellular acid phosphatase levels in macrophages, and secretion of toxins and (in some cases) hemolysin [2,4,23–25]. Furthermore, a phenotypically distinct form of *Nocardia* called L-phase variants or cell wall deficient variants is known to be induced within lungs and is involved in pathogenesis in in vivo animal models [26]. These forms, however, are not recovered from homogenates of infected lungs, making diagnosis difficult, and have been implicated in contributing to latency of disease [26].

In addition to issues related to immune evasion and diagnosis, strain identification also affects treatment outcomes. Large amounts of heterogeneity exist among different *Nocardia* species with genome sizes ranging from 6 to 10 million base pairs (Mbp) [27,28]. Virulence and antimicrobial susceptibilities of various pathogenic *Nocardia* species vastly differ from one another. The most recent classification distributes the clinically relevant *Nocardia* species into 13 antimicrobial susceptibility patterns [29]. *Nocardia farcinica* tends to be a more virulent species, intrinsically resistant to various antibiotics, including third-generation cephalosporins [18]. In addition, *N*. *cyriacigeorgica*, *N*. *nova*, and *N*. *pseudobrasiliensis* are considered major pathogenic species. The species also differ from each other in biochemical characteristics, such as their ability to utilize different carbon sources and hydrolyze different substrates. All these differences are used as criteria for species identification [18,30]. However, these techniques are laborious and require considerable expertise [31]. As a result, most clinical laboratories rarely identify *Nocardia* infections to the species level [32]. On account of the diverse antimicrobial susceptibilities associated with this genus, identification at species level is crucial for empirical treatment of infection with the appropriate antibiotic in the clinic [29].

Once diagnosed, treatment of nocardiosis is usually prolonged because of the risk of relapse [33,34]. Six to 12 months of antimicrobial therapy for immunocompetent patients and a minimum of 12 months of treatment for immunocompromised patients or those with CNS dissemination is often recommended [8]. In spite of the diverse susceptibility patterns among *Nocardia* species, all 13 patterns show sensitivity to the combination drug trimethoprim–sulfamethoxazole (TMP–SMX) and the more expensive, linezolid [29]. As a result, TMP–SMX is the treatment of choice for nocardial infections [29,35,36]. Imipenem, amikacin, and third-generation cephalosporins are also used, and combination therapy can yield better results [8,20].

## Antimicrobial resistance in *Nocardia*: Is it too late to prevent?

Resistance to antibiotics is a pressing problem and one of the biggest global threats facing the healthcare industry [37]. *Nocardia* species possess various patterns of intrinsic resistance to antibiotics, as mentioned above, and TMP–SMX is usually the treatment of choice. In addition to being used as a combination drug for long term treatment, TMP–SMX is also commonly used as a prophylactic agent at low doses to prevent *Nocardia* and *Pneumocystis jirovecii* infections in immunocompromised individuals [34,35,38]. However, this extended antibiotic regime and low dose exposure provide a greater opportunity for the evolution of resistance.

Unfortunately, resistance to TMP–SMX is already rampant. 42% of 765 isolates of *Nocardia* submitted to the CDC between 1995 and 2004 showed TMP–SMX resistance [39]. Similar levels of TMP–SMX resistance were also seen among 157 isolates from Canada [40]. Similarly, breakthrough infections in immunocompromised individuals receiving TMP–SMX prophylaxis are also being observed [34,35].

TMP–SMX inhibits the folate biosynthesis pathway in bacteria. Because these are two of the earliest antibiotic compounds with broad-spectrum efficacy to be administered to humans, resistance to these drugs is fairly common and well characterized in clinically important bacteria [41]. Resistance is often attributed to mutations or regulatory changes in target enzymes, efflux pumps, and acquired resistance via horizontal gene transfer [41]. The cause of resistance in clinical isolates of *Nocardia*, however, is understudied.

## What is known about the genetic basis of antimicrobial resistance in clinical *Nocardia* isolates?

A study in 2015 by Valdezate and colleagues showed that out of 76 TMP–SMX-resistant patient isolates of *Nocardia* belonging to 12 species, 75 carried Class 1 and/or Class 3 integrons, which are mobile elements associated with antimicrobial resistance [42,43]. In addition to carrying plasmid-borne variants of dihydropteroate synthase (DHPS) and dihydrofolate reductase (DHFR) (which are folate biosynthetic pathway enzymes targeted by SMX and TMP, respectively) the strains also carried genes encoding efflux pumps, as well as β-lactamases, aminoglycoside modifying enzymes, RNA methylases, and ribosomal protection proteins that are implicated in resistance to β-lactams, aminoglycosides, macrolides, and tetracyclines, respectively [43]. Although some isolates containing genetic determinants of resistance exhibited susceptible phenotypes, potentially owing to gene silencing or lack of functionality [43], these findings are alarming because they suggest that *Nocardia* isolates are capable of and already have acquired mobile elements carrying resistance conferring alleles that can spread rapidly via horizontal gene transfer. Although not much more is known about the genetic basis of resistance in *Nocardia*, these findings highlight the necessity to use caution in clinical settings during diagnosis and treatment of *Nocardia* infections.

## In vitro evolution of *Nocardia* to TMP–SMX

In the absence of data identifying de novo mutations that contribute to resistance in vivo, experimental evolution in vitro under the selective pressure from antibiotics can be used to recapitulate the paths leading to antimicrobial resistance in bacteria [44–46]. Recently, our group conducted in vitro experimental evolution to adapt susceptible clinical isolates of *N*. *nova* and *N*. *cyriacigeorgica* to the treatment of choice, TMP–SMX [28]. To our knowledge, this is the first study of its kind to identify the genetic basis of de novo resistance to TMP–SMX in *Nocardia*. Not surprisingly, mutations were seen within genes encoding DHFR and DHPS. Some of those mutations were identical to mutations implicated in resistance in other bacterial species like *Escherichia coli* and were involved in substrate or inhibitor binding. In addition to mutations affecting enzymes targeted by these drugs, changes were also seen in regulatory regions of genes encoding the folate pathway, which led to up-regulation. This resistance mechanism of overexpression of folate biosynthesis genes to increase flux through the pathway has also been observed in *Plasmodium falciparum* [47] and *E*. *coli* [48].

Interestingly, in addition to identifying known mechanisms of TMP–SMX resistance in *Nocardia*, this organism was able to achieve resistance via an as yet uncharacterized process. A homolog *(folP2)* of the gene encoding DHPS (*folP*) exists in most actinomycetes including *Nocardia* and *Mycobacterium*. Although considered to be nonfunctional and unable to serve as a bypass for DHPS in *Mycobacterium* [49], 8 out of 10 *Nocardia* strains evolved to TMP–SMX had acquired mutations in *folP2* [28], suggesting a role for this homolog in resistance. While sequence data of clinically TMP–SMX resistant *Nocardia* strains is lacking, this study provides potential biomarkers for diagnostic purposes. With next generation sequencing technology becoming cheaper and more easily accessible, we are moving closer to sequencing-based diagnostics for identification of infecting agents as well as their antimicrobial susceptibility profiles. Having prior knowledge of potential alleles involved in resistance will facilitate this process.

## Concluding remarks

The genus *Nocardia* encompasses a diverse group of species. While the incidence of nocardial disease is increasing and so is the number of species being identified [50], there is debate about the use of the phrase “emerging pathogen” for this organism [51]. It may be argued that modern molecular biology tools have improved diagnosis of this elusive pathogen and enabled finer species level identification, giving increased recognition to this well-established, rather than emerging, genus as an agent of infection. Conversely, information obtained from whole genome sequencing of at least one *N*. *cyriacigeorgica* genome suggests that this organism is on the path of an ongoing adaptation from an environmental bacterium to an emerging pathogen [52]. In either case, it is clear that accurate diagnosis and timely treatment of nocardial infections are crucial because undiagnosed and latent infections have the ability to spread from primary sites to more sensitive regions in the body that can be fatal. Development of PCR based diagnostic assays for early diagnosis of infection and rapid antimicrobial susceptibility testing to identify appropriate treatment options should be considered a priority for this pathogen. As rapid molecular diagnostic technology continues to improve it is likely that other organisms, like *Nocardia*, may soon be joining an extended pantheon of medically important pathogens.
